# Effect of filler size and filler loading on wear of experimental flowable resin composites

**DOI:** 10.1590/1678-7757-2016-0652

**Published:** 2018-01-16

**Authors:** Koichi Shinkai, Yoshihisa Taira, Shiro Suzuki, Satoki Kawashima, Masaya Suzuki

**Affiliations:** 1 The Nippon Dental University School of Life Dentistry at Niigata, Department of Operative Dentistry, Niigata, Japan; 2 University of Alabama at Birmingham, School of Dentistry, Department of Clinical and Community Sciences, Birmingham, United States of America

**Keywords:** Dental restoration wear, Composite resins, Dentistry, operative, In vitro techniques

## Abstract

The relationship between wear resistance and filler size or filler loading was clarified for the universal resin composite; however, their relationship in flowable resin composites has not been clarified.

**Objectives:**

The purpose of this study was to investigate the effect of filler size and filler loading on wear of experimental flowable resin composites by using a cyclic loading device.

**Material and Methods:**

Nine experimental flowable resin composites consisting of three different sizes (70, 200 and 400 nm) and loading (50, 55 and 60 wt%) of filler were prepared. Bowl-shaped cavities were prepared on a flat surface of ceramic blocks using a No. 149 regular cut diamond point. The cavities were treated with a silane coupling agent and an all-in-one adhesive and then filled with each experimental flowable resin composite. The restored surfaces were finished and polished with a 1500-grit silicon carbide paper. The specimens were subjected to an *in vitro* two-body wear test using a cyclic loading device. The localized worn surfaces were evaluated at 10,000, 20,000, 30,000, and 40,000 cycles using a computer-controlled three-dimensional measuring microscope (n=5). The volumetric wear loss of the materials was calculated automatically by the equipment. Data were statistically analyzed with two-way ANOVA and *post hoc* Tukey test.

**Results:**

Two-way ANOVA showed that the filler size significantly influenced wear volume (p<0.003), but the filler loading did not have a significant effect (p>0.05). A *post hoc* Tukey test detected significant differences in filler size between 70 nm and 400 nm, and 200 nm and 400 nm (p<0.007).

**Conclusion:**

The experimental flowable resin composite containing a mean filler size of 400 nm exhibited significantly lower wear resistance in two-body wear compared with those containing mean filler sizes of 200 nm or 70 nm.

## Introduction

First generation flowable resin composites were only used in low stress bearing areas because of inferior physical properties^1^. Seemann, et al.^15^ (2011) reported that the physical properties of flowable resin composites were improved by increasing the filler particle concentration and modifying the filler size^15^. In addition, flowable resin composites are easy to use to fill cavities using a direct-application-syringe. Owing to such improvements in physical and handling properties, the application of flowable resins was expanded to posterior restorations. However, posterior restorations are considerably stressed by cyclic loading during mastication. Consequently, resin composite restorations are subjected to occlusal wear over time^12^ ^,^ ^14^. *In vitro* and *in vivo* studies have reported the wear resistance of universal resin composites, whose wear resistance was considerably improved by adding variously sized filler particles^3^ ^,^ ^6^ ^,^ ^9^ ^-^ ^11^ ^,^ ^20^ ^,^ ^21^. Although wear of universal hybrid resin composites is no longer considered a major clinical problem^4^ ^,^ ^11^ ^,^ ^13^, other research concluded that the wear resistance of flowable resin composites for posterior restorations was quite limited^2^ ^,^ ^14^. On the other hand, Sumino, et al.^18^ (2013) reported that the localized wear and flexural properties of the flowable resin composites tested were equivalent to those of universal resin composites produced by the same manufacturers^18^. Thus, the wear resistance of flowable resin composite is still a controversial area.

Our previous study^17^, which examined three- and two-body wear values of flowable resin composites for posterior restoration using a mechanical loading device, demonstrated that the wear resistance of the flowable resin composite containing nanofillers or spherical fillers was equivalent to that of a universal resin composite used as a control. The study also suggested that the size and shape of fillers in the flowable resin composite might influence both three- and two-body wear resistances. For the universal resin composite, the relationship between wear resistance and filler size or filler loading was clarified by many studies^2^ ^,^ ^5^ ^,^ ^8^ ^,^ ^9^ ^,^ ^11^ ^,^ ^18^. However, their relationship in flowable resin composites has not been clarified. The purpose of this study was to examine the effect of the size and loading of filler on the two-body wear resistance of experimental flowable resin composites using an *in vitro* wear simulator. The null hypothesis was that filler size and loading would not influence the two-body wear resistance of experimental flowable resin composites.

## Material and methods

**Figure 1:**
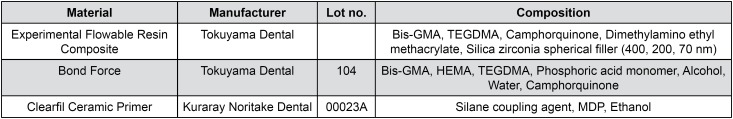
Materials used in this study

**Figure 2:**
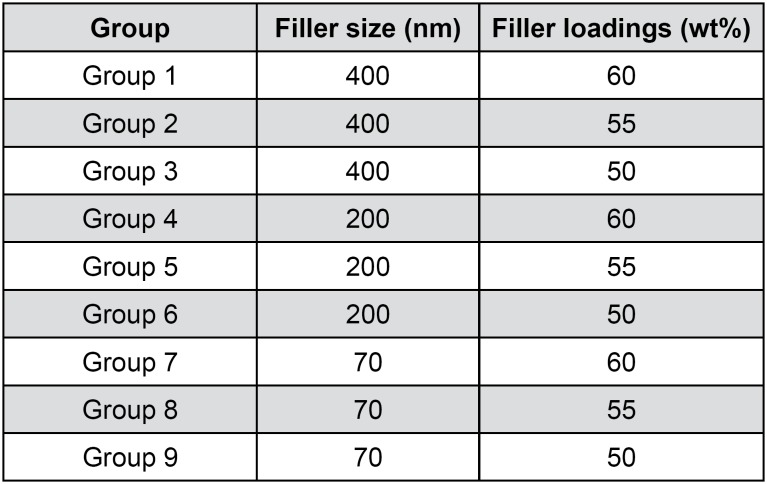
Experimental flowable resin composite used in this study

**Figure 3:**
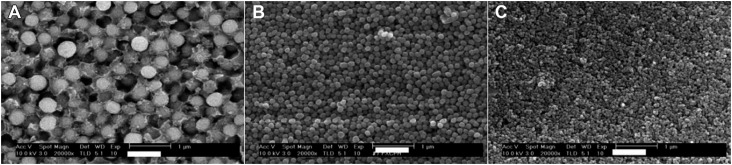
Scanning electron microscopy (SEM) photographs of fillers containing experimental flowable resin composites (magnification 20,000×) (A) Mean filler diameter of 400 nm; (B) Mean filler diameter of 200 nm; (C) Mean filler diameter of 70 nm

The materials used in this study are presented in Figure 1. The experimental flowable resin composites were developed in collaboration with Tokuyama Dental, Tokyo, Japan. Nine experimental flowable resin composites consisting of different sizes and loadings of spherical fillers were used. The size and loading of the spherical filler in each group is shown in Figure 2. The experimental flowable resin composites used in this study are shown in Figure 3. A bonding agent (Bond Force, Tokuyama Dental Corp., Tokyo, Japan) and a ceramic primer (Clearfil Ceramic Primer, Kuraray Noritake Dental Inc., Tokyo, Japan) were used for bonding between a ceramic cavity and experimental flowable resin composites.

### Specimen preparation

A bowl-shaped cavity (4 mm diameter, 2 mm depth) was prepared in the center of the flat surface of a ceramic block (Vitabloc Mark II, Ivoclar Vivadent Inc., NY, USA) with a no. 149 regular-cut diamond point under 300,000 rpm with copious irrigation. The cavities were treated with 40% phosphoric acid (K-Etchant, Kuraray Noritake Dental Inc., Tokyo, Japan) for 10 s, and then water-sprayed and air-blown for 5 s each. The cavities were treated with the ceramic primer. A bonding agent (Bond Force, Tokuyama Dental Corp., Tokyo, Japan) was applied and photopolymerized for 10 s. The cavities were restored with the experimental flowable resin composites by using a two-layer incremental technique (*n*=5). Each layer was photopolymerized for 30 s with a light-curing unit (Candelux, Morita Corp., Tokyo, Japan). The second layer was slightly overfilled. The specimens were stored in a humidity-controlled (95%) device at 37°C for 48 h. The restored surfaces of the specimens were finished and polished by wet-grinding with a 1500-grit silicon carbide paper (*n*=5).

### Two-body wear testing

The specimens were fixed to a stainless cup with an acrylic resin, and the cups were mounted on a cyclic loading device (Ito Electric Construction, Niigata, Japan). The resin restorations were subjected to a two-body wear test, in which a conical ceramic (aluminum nitride) stylus was used to apply a cyclic compressive load of 75 N to the surface of each restoration at a rate of 120 contacts/min.

### Measurement of two-body wear of restorations

The localized worn surfaces of the restorations were scanned at 10,000, 20,000, 30,000, and 40,000 cycles with a computerized three-dimensional microscope (STM6DF, Olympus Corp., Tokyo, Japan). The volume of the worn area was obtained using a computer software package associated with the microscope.

### Microscopic observation of the worn surfaces

The worn surfaces of a representative specimen in each group after 40,000 cycles were observed using a scanning electron microscope (SEM, S-800, Hitachi Corp., Tokyo, Japan) at ×40 and ×5,000 magnification.

### Statistical analysis

Data were statistically analyzed with two-way analysis of variance (ANOVA) and *post hoc* Tukey test to specify the influence of the size and loading of filler on the wear volume after each cycle at a 0.05 significance level. In addition, the statistical differences in wear volume among respective groups after each cycle were determined using one-way ANOVA and *post hoc* Tukey test at a 0.05 significance level. Statistical analysis was carried out with the Ekuseru-Toukei 2010 software system (Social Survey Research Information Co., Ltd., Tokyo, Japan).

## Results

**Figure 4:**
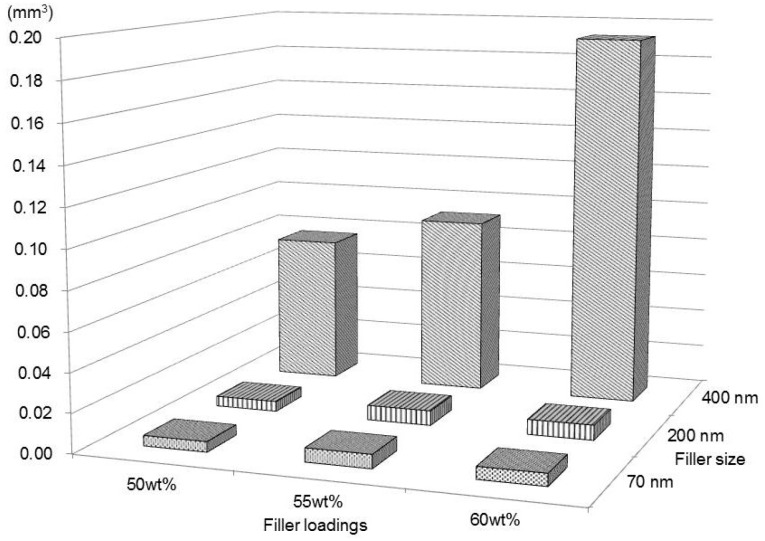
The correlation of the wear volumes between the two factors after 40,000 cycles

The mean wear volumes of each material at the respective wear cycles are presented in Table 1, and the correlation of the wear volumes between the two factors after 40,000 cycles is shown in Figure 4. Although all of them tended to increase gradually with the number of wear cycles, the increase in groups 1, 2, and 3 was considerably larger compared with those of the other groups. After all wear cycles, two-way ANOVA showed that the filler size significantly influenced wear volume (*p*<0.003), but the filler loading did not significantly affect wear volume (*p*>0.05), and a significant interaction between these factors was not recognized (*p*>0.05). The *post hoc* Tukey test for the factor of filler size revealed significant differences in wear volume between 70 nm and 400 nm, and 200 nm and 400 nm (*p*<0.007). However, there was no significant difference in wear volume between 70 nm and 200 nm filler size (*p*>0.05). After 20,000, 30,000 and 40,000 cycles, one-way ANOVA and the *post hoc* Tukey test revealed significant differences in wear volume among group 1 and the other groups except for groups 2 and 3 (*p*<0.035) (Table 1). However, there were no significant differences in wear volume among all the groups after 10,000 cycles (*p*>0.05) (Table 1).

**Table 1:** Wear volumes of materials at each wear cycle (Mean±SD, Unit: mm^3^)

Group	Wear cycle			
	10,000	20,000	30,000	40,000
Group 1	0.033±0.035^a^	0.085±0.083^b^	0.144±0.134^d^	0.192±0.162^f^
Group 2	0.024±0.037^a^	0.047±0.061^bc^	0.069±0.104^de^	0.091±0.133^fg^
Group 3	0.011±0.015^a^	0.030±0.041^bc^	0.050±0.065^de^	0.076±0.100^fg^
Group 4	0.001±0.001^a^	0.003±0.003^c^	0.005±0.005^e^	0.008±0.008^g^
Group 5	0.001±0.002^a^	0.004±0.003^c^	0.005±0.005^e^	0.008±0.007^g^
Group 6	0.001±0.001^a^	0.003±0.003^c^	0.004±0.004^e^	0.005±0.005^g^
Group 7	0.001±0.001^a^	0.002±0.003^c^	0.004±0.007^e^	0.006±0.008^g^
Group 8	0.001±0.001^a^	0.003±0.003^c^	0.005±0.004^e^	0.007±0.005^g^
Group 9	0.001±0.001^a^	0.002±0.002^c^	0.004±0.004^e^	0.005±0.005^g^

Within the same column, Mean±SD with different capital superscript letter are statistically different (p<0.05)

**Figure 5:**
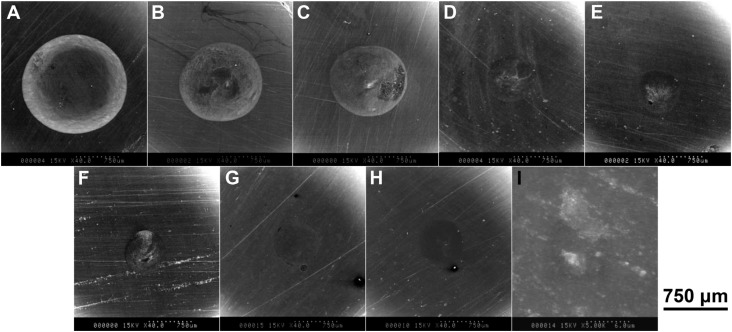
Representative scanning electron microscopy (SEM) photographs of the materials after 40,000 two-body wear cycles (×40 magnification) (A) Group 1; (B) Group 2; (C) Group 3; (D) Group 4; (E) Group 5; (F) Group 6; (G) Group 7; (H) Group 8; (I) Group 9 (A), (B) and (C), large indentation produced by localized wear was observed on each specimen.; (D), (E) and (F), the size of the indentations was small, but their outline was unclear; (G), (H) and (I), the specimens showed indistinct indentations

**Figure 6:**
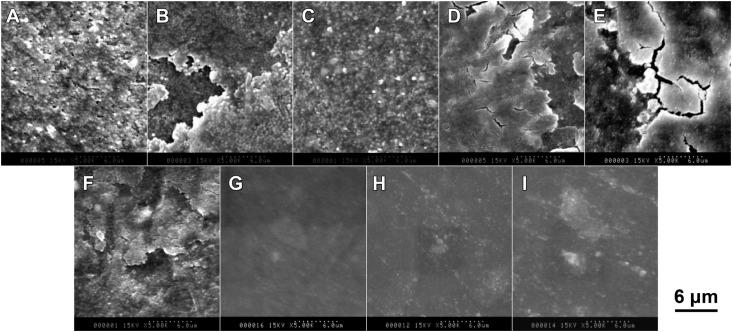
Representative scanning electron microscopy (SEM) photographs of worn material surfaces after 40,000 two-body wear cycles (×5,000 magnification) (A) Group 1; (B) Group 2; (C) Group 3; (D) Group 4; (E) Group 5; (F) Group 6; (G) Group 7; (H) Group 8; (I) Group 9 (A), (B) and (C), Individual filler particles can be detected on the worn surface.; (D), (E) and (F), a defect with cracks was observed on the specimens.; (G), (H) and (I), the worn surfaces on the specimens were extremely homogeneous

Figure 5 shows representative SEM photographs (×40) of each material after 40,000 cycles. A large indentation produced by localized wear was observed on each specimen from groups 1, 2 and 3 (400 nm filler size groups). On the specimens from groups 4, 5 and 6 (200 nm filler size groups), the size of the indentations was small, but their outline was unclear. On the other hand, the specimens from groups 7, 8 and 9 (70 nm filler size groups) showed indistinct indentations. Figure 6 shows that representative T Individual filler particles can be detected on the worn surface of groups 1, 2 and 3. A defect with cracks was observed on the specimens of groups 2, 4, 5 and 6, while the worn surfaces on the specimens of groups 7, 8 and 9 were extremely homogeneous compared with the other specimens.

## Discussion

Filler size and filler loading, hardness of polymerized resin matrix, and adhesive strength between the filler and resin matrix are factors influencing the wear resistance of resin composites. The effect of filler size or filler loading on the wear resistance of resin composites has been investigated in *in vitro* studies^2^ ^,^ ^7^ ^,^ ^9^ ^,^ ^10^ ^,^ ^18^. Johnsen, et al.^9^ (2011) examined the effect of filler loading (wt%) and particle size on surface roughness and wear resistance under a wear model closer to a clinical situation using the polisher/grinder machine. They suggested that the most wear resistant experimental resin composite should consist of medium filling loading (75%) but that filler particle size is not as critical as reported in the past. On the other hand, Sumino, et al.^18^ (2013) reported that the localized wear value of the flowable resin composite containing extra-small sized fillers (0.016 and 0.2 μm) was significantly lower than those containing larger sized fillers (3 and 20 μm). Our previous study^17^ showed that a flowable resin composite containing fillers of 0.8 μm mean size demonstrated significantly better localized wear resistance compared with that containing fillers of 3 μm mean size. Thus, the inclusion of a smaller sized filler may be advantageous compared with a large sized filler for localized wear resistance of flowable resin composites. These previous studies investigated and compared the wear resistance of flowable resin composites using some marketed products. Since the compositions of the resin matrix provided in marketed flowable resin composites are different among respective manufacturers, the hardness of the polymerized resin matrix for each marketed resin composite may be different, and the differences seem to affect the wear resistance of the resin composite. Therefore, experimental flowable resin composites consisting of different sizes and fillers with the same resin matrix were used in this study.

The results of two-way ANOVA showed that the filler size had a relationship with the wear resistance after all wear cycles. However, the filler loading had no relationship after all wear cycles. It was interesting that the resin composite containing larger sized fillers (400 nm) exhibited significantly greater amounts of wear volume compared with that containing smaller sized fillers (200 nm or 70 nm) after all wear cycles, regardless of filler loading. In addition, the wear volume of the resin composite with 400 nm fillers increased as the filler loading increased. Neither filler size nor filler loading affected the two-body wear resistance of the experimental flowable resin composite containing 200 nm or 70 nm sized filler particles. The SEM images in Figures 4 and 5 show numerous protruded spherical fillers on the specimens from groups 1, 2 and 3, and catastrophic defects with cracks in groups 1 to 6, but there are no defects in groups 7, 8 and 9. Therefore, these protruded larger fillers might have experienced greater friction with the stylus tip that might provoke filler exfoliations and crack formation during wear testing that led to accelerated wear, as shown in previous studies^7^ ^,^ ^9^ ^,^ ^10^. Moreover, it was speculated that the exfoliated fillers might have acted as an abrasive medium.

Our previous study using the same wear simulator demonstrated that the two-body wear value of the flowable resin composite containing large sized fillers was significantly higher than those containing smaller sized fillers^17^. Moreover, the other previous study also demonstrated that the flowable resin composite containing large fillers showed significantly deeper defects for cyclic impact loadings compared with those containing small fillers^16^. From the results of these studies, it was speculated that a remarkable surface degradation of a flowable resin composite containing large fillers at the stylus contact area might be caused by micro-crack formation at the filler/matrix interface and additional micro-fractures in the resin matrix. The wear simulator used in our study uses grinding movement and impact loading. Similar phenomena creating an early surface degradation might have occurred in the experimental flowable resin composite containing larger sized fillers in this study. However, micro-cracks may hardly be grown on those containing smaller sized fillers; as a result, a smooth surface texture of the polymerized resin composite seems to be maintained during the two-body wear testing.

The adhesion between filler and polymerized resin matrix could also be related to the wear resistance of flowable resin composites. Tamura, et al.^19^ (2013) reported that the filler loading is directly related to the occlusal wear of experimental resin composites containing four types of filler particles, including non-porous spherical silica, porous spherical silica, porous spherical zirconium silicate, and irregularshaped silica. However, the mechanical properties tested (flexural strength, elastic modulus, and Vickers hardness) showed no correlation with the occlusal wear. The study showed that the experimental resin composites containing porous spherical fillers exhibited significantly higher wear resistance compared with those containing non-porous spherical fillers. They speculated that the porous surface texture would produce a strong bond between the filler particles and resin matrix because of the mechanical retention of the resin matrix that penetrated into the tiny concavities. From the results of the simulated occlusal wear test, they mentioned that the bonding between the filler particles and resin matrix dominantly influenced the occlusal wear, and indicated that the occlusal wear could not be directly influenced by the mechanical properties. The average filler particle size containing these resin composites was 1.7 to 2.5 μm, which was much larger compared with the experimental flowable resin composites used in this study. The protruded large filler particles might sustain greater friction with the stylus tip during occlusal wear testing.

From the results of our study, the null hypothesis that filler size and filler loading would not influence the two-body wear resistance of experimental flowable resin composites was partially rejected. Up to 40,000 wear cycles, the filler size significantly influenced wear volume, but the filler loading did not have a significant effect.

## Conclusion

The experimental flowable resin composite containing a mean filler size of 400 nm exhibited significantly lower wear resistance in two-body wear compared with those containing mean filler sizes of 200 nm or 70 nm.
